# Early efficacy and safety of spinal endoscopy assisted anterior cervical discectomy and fusion in the treatment of cervical spondylotic myelopathy

**DOI:** 10.3389/fonc.2026.1678009

**Published:** 2026-02-18

**Authors:** Haijun Ma, Lijuan Zhan, Mingkui Shen, Zhongxin Tang, Jun Tan

**Affiliations:** 1 Department of Mini-invasive Spinal Surgery, The Third People’s Hospital of Henan Province, Zhengzhou, Henan, China; 2 Department of Neurology, People’s Hospital of Zhengzhou, Zhengzhou, Henan, China

**Keywords:** anterior cervical discectomy and fusion, cervical spondylotic myelopathy, endoscopy, percutaneous full-endoscopic, spinal endoscopy

## Abstract

**Purpose:**

To introduce a new surgical approach for spinal endoscopy assisted anterior cervical discectomy and fusion (Endo-ACDF) in treating cervical spondylotic myelopathy (CSM) and to report the clinical results after a 2-year follow-up.

**Methods:**

The clinical data of 123 CSM patients who underwent ACDF from February 2020 to February 2022 were retrospectively analyzed. They were divided into two groups: Open-ACDF and Endo-ACDF, based on different surgical methods. Baseline data, hospitalization duration, operation time, intraoperative blood loss, postoperative drainage, and postoperative Japanese orthopedic association (JOA) score, neck disability index (NDI), visual analogue scale (VAS) scores and imaging results were compared between the two groups.

**Results:**

There was no statistically significant difference between the baseline data of the two groups (*P* > 0.05). The intraoperative estimated blood loss and postoperative drainage in the Endo-ACDF group were less than those in the Open-ACDF group (*P* < 0.05). The postoperative JOA score, NDI, VAS, height of the adjacent vertebral body (HAVB), and cervical lordosis angle (CLA) in both groups were significantly improved compared to the preoperative period, with statistically significant differences (*P* < 0.05). Compared with the Open-ACDF group, postoperative CLA and HAVB were significantly improved in the Endo-ACDF group, with better clinical outcomes at 1 year postoperatively (*P* < 0.05). At the last follow-up, HAVB remained higher in the Endo-ACDF group (*P* < 0.05), but there was no difference in CLA between the two groups (*P* > 0.05).

**Conclusion:**

Endo-ACDF combines the endoscopic system with ACDF technology for treating CSM, demonstrating clinical efficacy comparable to Open-ACDF. Compared to Open-ACDF, Endo-ACDF offers a clearer surgical field, improved intraoperative hemostasis, and reduced intraoperative blood loss and postoperative drainage.

## 1 Introduction

Cervical spondylotic myelopathy (CSM) is the most severe form of cervical spondylosis, characterized by cervical intervertebral disc degeneration that compresses the spinal cord or disrupts its blood supply, resulting in sensory, motor, and sphincter dysfunctions (1–3). Surgical intervention remains the most direct and effective treatment for CSM, including anterior, posterior, or combined anterior–posterior approaches (4, 5). In 1955, Smith and Robinson first introduced anterior cervical discectomy and fusion (ACDF) for the treatment of cervical spondylosis (6, 7). ACDF has gradually evolved into a standard procedure for CSM, effectively improving neurological and spinal symptoms, restoring intervertebral height and cervical curvature, and maintaining cervical stability (8). However, conventional ACDF is limited by a restricted operating space, narrow visual field, and challenging hemostasis, which hinder clear identification of anatomical structures and complicate decompression of the spinal cord and nerve roots (9, 10). In recent years, continuous advancements in spinal endoscopic technology have gradually transformed spinal surgery into an “endoscopic” discipline, offering new therapeutic options for cervical degenerative diseases (11, 12).

In recent years, our center has adopted spinal endoscopy–assisted anterior cervical discectomy and fusion (Endo-ACDF) for the treatment of CSM, achieving satisfactory surgical outcomes and a lower incidence of complications. Therefore, this retrospective study compared the clinical efficacy of Endo-ACDF and open ACDF in patients with CSM to further elucidate the therapeutic advantages of Endo-ACDF.

## 2 Materials and methods

### 2.1 Inclusion criteria

Inclusion criteria were as follows: (1) clinical signs and symptoms of cervical spinal cord compression, including numbness or weakness in the upper and/or lower limbs, hyperreflexia, and positive pathological reflexes; (2) complete X-ray, CT, and MRI data confirming single-level spinal cord compression; (3) diagnosis of CSM established based on medical history, clinical presentation, and imaging findings; (4) compression located on the ventral aspect of the spinal cord; (5) lack of improvement after 3–6 months of conservative, non-surgical treatment.

### 2.2 Exclusion criteria

Exclusion criteria were as follows: (1) isolated cervical radiculopathy without evident signs of spinal cord compression; (2) conditions unsuitable for anterior surgical approaches, including ossification of the posterior longitudinal ligament, congenital or degenerative spinal stenosis, or posterior spinal cord compression; (3) involvement of two or more cervical segments; (4) history of cervical fracture, prior cervical or tracheal surgery, infection, or tumor; (5) poor general condition, such as severe osteoporosis or coagulopathy, rendering surgery intolerable; (6) incomplete follow-up data.

### 2.3 Patient selection

This study was approved by the Medical Ethics Committee of the Third People’s Hospital of Henan Province (Approval No.: 2020-012). Owing to the retrospective design and anonymized patient data, the requirement for written informed consent was waived by the Ethics Committee in accordance with the Declaration of Helsinki. All procedures were performed by surgeons with 5–10 years of experience in degenerative spine surgery. A total of 123 patients who met the inclusion criteria were enrolled between February 2020 and February 2022. Patients were divided into the Endo-ACDF group (n = 65) and the Open-ACDF group (n = 58) based on the surgical approach.

### 2.4 Open-ACDF

The ACDF procedure has been described previously (13). After induction of general anesthesia, the patient was placed in the supine position with the shoulders elevated and the neck in a neutral position. Both upper limbs were fixed and gently extended with appropriate distal traction. Under X-ray fluoroscopic guidance, the cervical spine was exposed through a standard right-sided anterior approach to the anterior cervical fascia. The carotid tubercle adjacent to the C6 vertebral body was palpated to preliminarily locate the target vertebral segment. A localization needle was inserted into the target vertebral body, and X-ray fluoroscopy was performed to confirm the surgical level. The anterior longitudinal ligament was excised under direct visualization. The annulus fibrosus was incised, and disc material was removed up to the posterior margin of the vertebral body using nucleus rongeurs and curettes. A parallel spreader was inserted at the posterior edge of the vertebral body to widen the intervertebral space. The Caspar distractor was then tightened to maintain the intervertebral height. Residual disc material was removed to expose the posterior margin of the vertebral body and the posterior longitudinal ligament. The bony ridges at the posterior margin of the vertebral body were removed using a laminectomy rongeur or a high-speed grinding drill. Protruding disc fragments extending into the spinal canal were carefully removed up to the uncovertebral joints bilaterally. After determining the appropriate intervertebral height with a trial spacer, an interbody cage filled with autologous bone was inserted into the intervertebral space. A titanium plate of appropriate length was then fixed to the anterior vertebral bodies with monocortical screws, followed by tightening the locking screws.

### 2.5 Endo-ACDF

After induction of general anesthesia, the patient was placed in the supine position with both upper limbs gently retracted and fixed distally. C-arm X-ray fluoroscopy was used to localize the surgical level, and the incision site was marked on the skin surface. The surgical field was sterilized using standard aseptic techniques. A waterproof drape was placed, and a sterile waterproof sheet with an integrated drainage system was applied over the anterior cervical region to prevent fluid leakage and contamination during the water-mediated endoscopic procedure. The pneumatic free arm was fixed to the surgical table, and the endoscope was attached to the sheath, which was then secured to the arm. The power grinding drill, bipolar electrocoagulation hemostatic forceps, unipolar high-frequency electrosurgical knife, and bipolar spherical radiofrequency electrodes are connected to their respective ports.

Under air-mediated endoscopic guidance, a 3-cm transverse incision was made on the right side of the neck to expose the cervical platysma. The sternocleidomastoid and strap muscles were gently separated and retracted superiorly and inferiorly along their deep surfaces, and the superficial cervical fascia was carefully incised. The anterior border of the vertebral body was then bluntly dissected. Bipolar coagulation forceps were used for hemostasis, and the anterior fascia was incised with a monopolar electrocautery to expose the anterior surface of the intervertebral disc and adjacent vertebral body. A localization pin was then inserted into the proximal vertebral body, and fluoroscopy was used to confirm the correct surgical level. Finally, the Caspar distractor pins were inserted, and the Caspar distractor was secured. Under air-mediated endoscopic visualization, the annulus fibrosus was incised up to the uncovertebral joints bilaterally. The intervertebral disc material was removed using pituitary rongeurs or curettes, exposing the posterior border of the vertebral body and the uncovertebral joints on both sides. The intervertebral space was then distracted using a Caspar distractor.

The endoscope was adjusted to a water-mediated view. A high-speed grinder drill was used to remove bony ridges at the posterior vertebral margin, exposing the posterior annulus fibrosus and posterior longitudinal ligament. The posterior longitudinal ligament was gently probed with a nerve dissector to expose the dural sac. Portions of the annulus fibrosus and posterior longitudinal ligament were removed using a fine laminectomy rongeur. The dural sac was re-examined with a nerve dissector to confirm complete decompression. Finally, residual cartilaginous endplates were carefully removed under water-mediated endoscopic visualization to avoid injury to the bony endplates.

**Figure 1:**
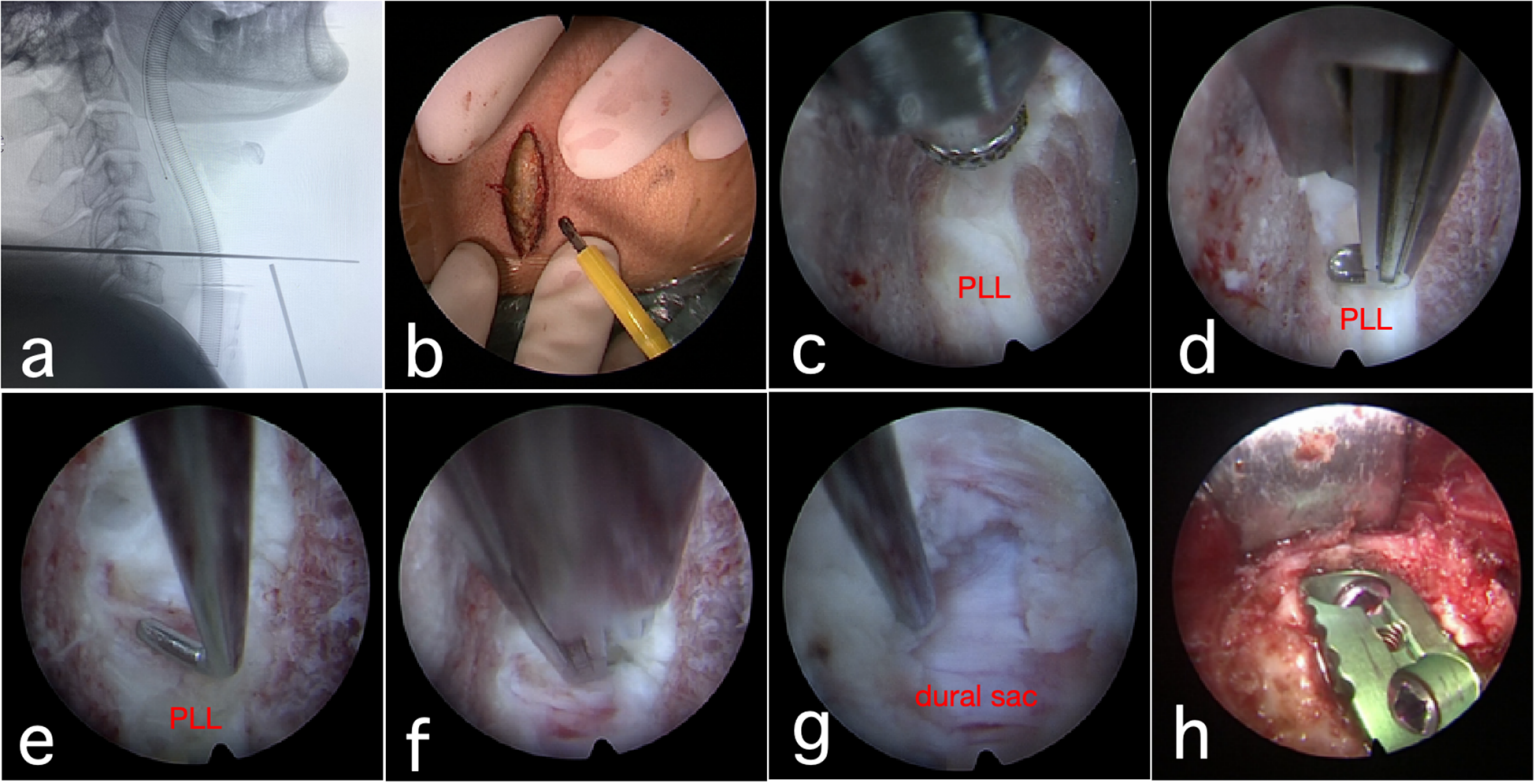
Intraoperative endoscopic diagrams. **(a)**: Determination of surgical segments using C-arm X-ray fluoroscopy; **(b)**: Intraoperative incisions under air-mediated endoscopic view; **(c)**: The upper and lower endplates were treated with a grinding drill in water medium; **(d)**: The posterior vertebral margin was treated with a thin laminectomy rongeur; **(e)**: Probing the posterior longitudinal ligament and dural sac using a nerve stripper; **(f)**: Removal of the posterior longitudinal ligament using a thin laminectomy rongeur; **(g)**: The dural sac was re-explored with a nerve stripper to confirm adequate decompression; **(h)**: A zero-profile cage filled with autogenous and artificial bone was placed. PLL, posterior longitudinal ligament.

The endoscope was switched to an air-mediated view to further confirm complete decompression. A zero-profile interbody cage filled with autologous and synthetic bone graft was inserted, the Caspar distractor was removed, and screw fixation was completed. The positions of the cage and screws were confirmed under fluoroscopy. No active bleeding was observed. The surgical site was irrigated, drains were placed, and the incision was closed in layers (**Figure 1**; **Supplementary Video**).

### 2.6 Postoperative treatment

**Figure 2:**
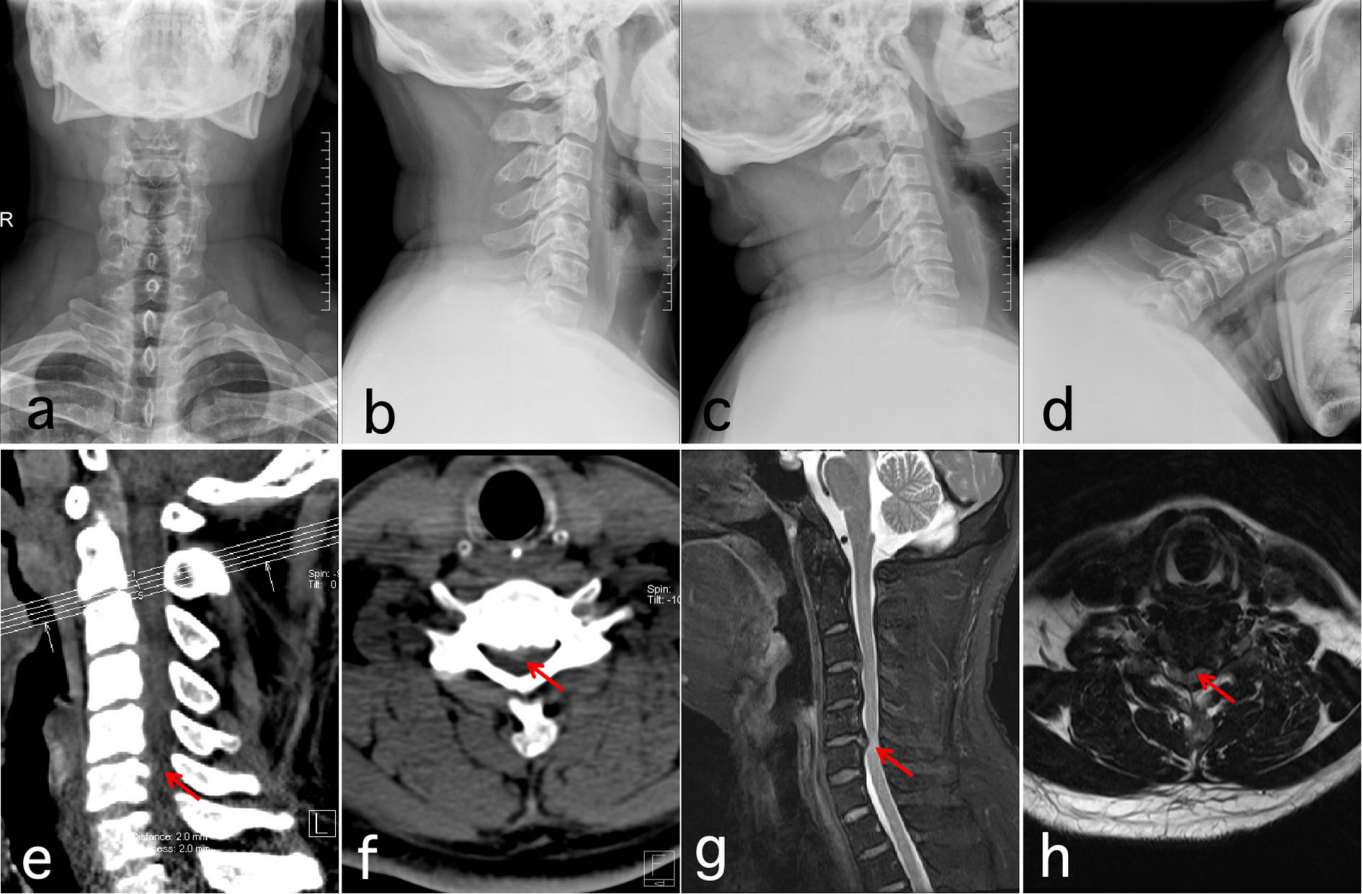
Images from a patient treated with Endo-ACDF for CSM. **(a-d)** Preoperative X-ray show cervical degeneration and straightening of the cervical physiological curvature; **(e-h)** Preoperative MRI and CT showed a herniated disc at the C5–6 segment with spinal stenosis and spinal cord compression.

**Figure 3:**
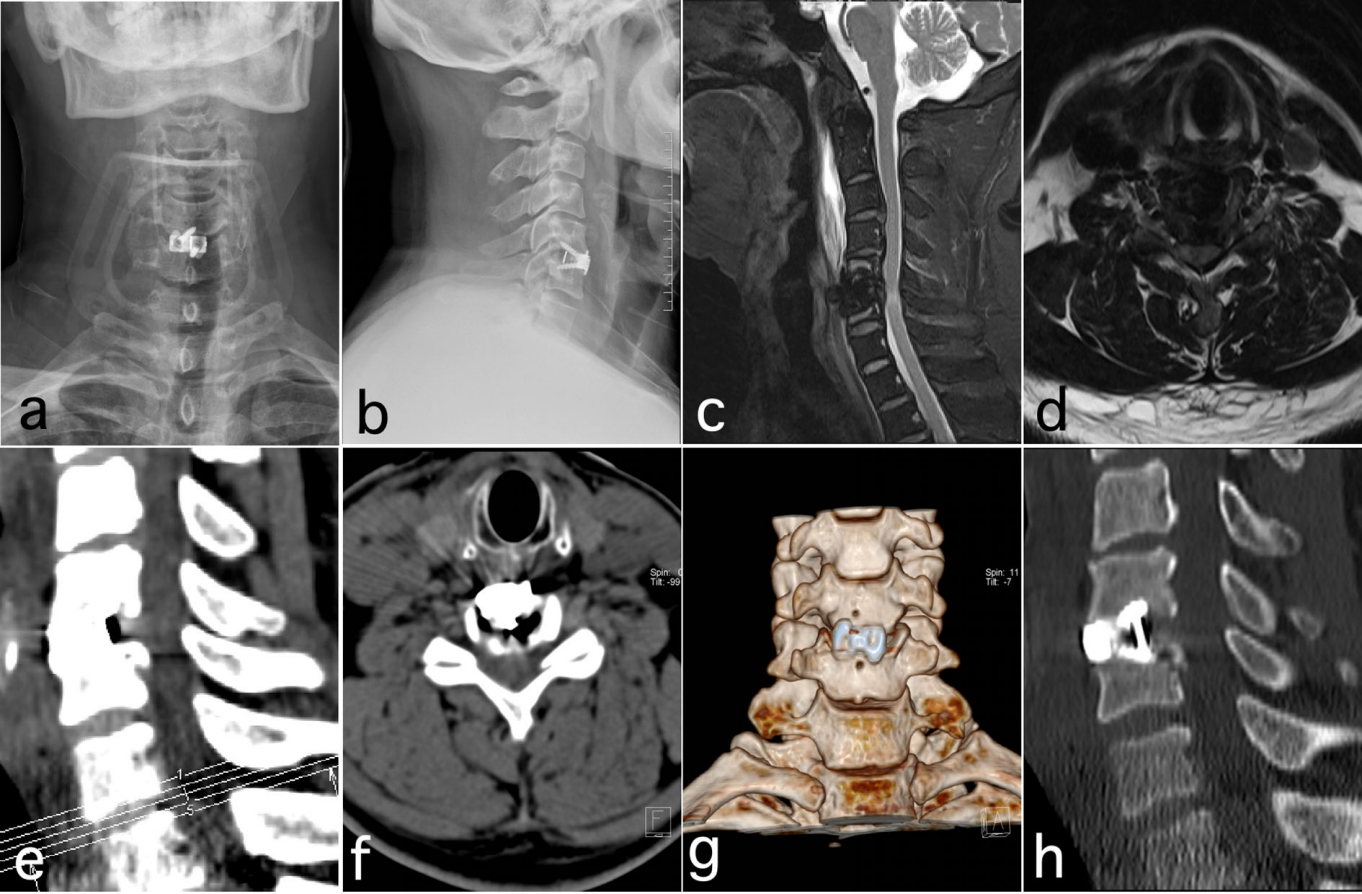
Postoperative review images of Endo-ACDF for CSM at 1 week. **(a, b)** X-ray shows the cage in a good position; **(c-f)** Postoperative MRI and CT showed effective decompression of the spinal cord and spinal canal; **(g, h)** Postoperative CT 3D reconstruction showed the cage in a proper position and at an appropriate depth.

To prevent surgical site infection (SSI), a second-generation cephalosporin was administered as follows: a single dose 30 minutes preoperatively, an additional dose intraoperatively if the operation lasted >3 hours or blood loss exceeded 1500 mL, and discontinued within 24 hours postoperatively. The drainage tube was removed when output was <5 mL within 1–2 days postoperatively, and patients were instructed to properly wear a cervical brace and mobilize early. Cervical X-ray, CT, and MRI were performed 1–3 days postoperatively to evaluate screw and cage positioning, bone graft placement, and spinal cord decompression (**Figures 2**, **3**). Postoperatively, patients were instructed to wear a cervical brace for 4–6 weeks and attend regular outpatient follow-up (14).

### 2.7 Clinical evaluation

Operation time, intraoperative blood loss, serum creatine phosphokinase (CPK) and C-reactive protein (CRP) levels, as well as postoperative drainage, were compared between the two groups (15). Postoperative outcomes were assessed using the Japanese Orthopaedic Association (JOA) score, the Neck Disability Index (NDI) score (16), and the visual analog score (VAS). Changes in intervertebral space height, cervical lordosis of the affected segments, and bone fusion were evaluated. Intraoperative blood loss was calculated by subtracting the irrigation fluid volume from the suctioned fluid, combined with the weight difference of gauze and surgical towels before and after surgery.

### 2.8 Image evaluation

**Figure 4:**
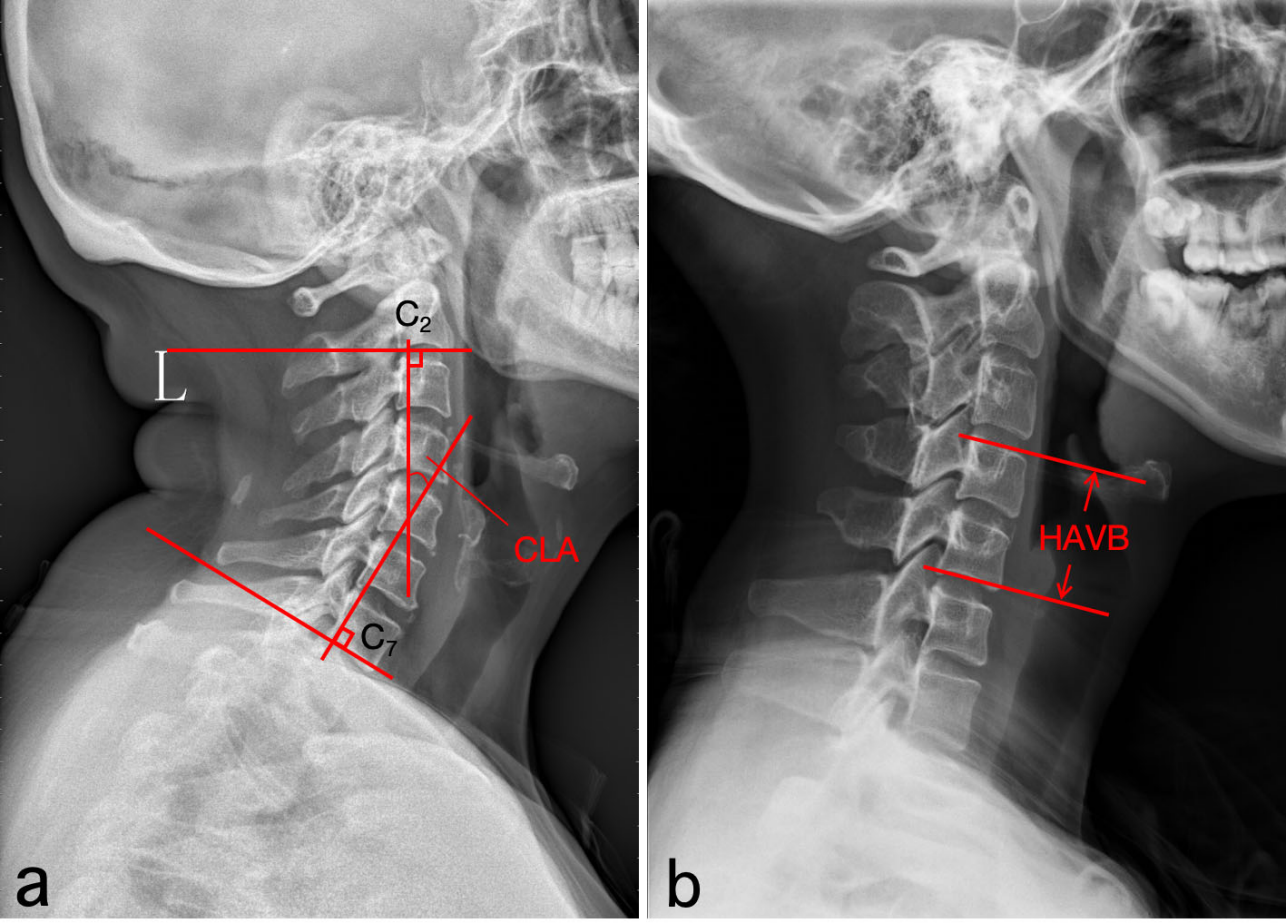
Preoperative and postoperative imaging evaluation. **(a)** cervical lordosis angle (CLA); **(b)** height of the adjacent vertebral body (HAVB).

Comparison of cervical lordosis angle (CLA), C2–C7 range of motion (ROM), and height of the adjacent vertebral body (HAVB) between preoperative measurements and the final follow-up. CLA: Defined as the acute angle formed between the vertical lines of the inferior endplates of the C2 and C7 vertebral bodies on lateral cervical spine radiographs. HAVB: Measured as the vertical distance between the superior endplate of the upper vertebral body and the inferior endplate of the lower vertebral body. ROM: Calculated as the difference between the maximum extension and maximum flexion angles of C2–C7 (**Figure 4**).

### 2.9 Statistical methods

SPSS (22.0 Inc., Chicago, IL) was used for statistical analysis. Normally distributed measurement data were expressed as mean ± standard deviation. An independent samples *t* test was used for comparisons between the two groups, and a paired *t* test was used for comparisons between preoperative and postoperative periods. Repeated measures ANOVA was used for comparisons between different time points. The chi-square test and Fisher’s exact probability method were used for categorical data. Differences were considered statistically significant at *P* < 0.05.

## 3 Results

### 3.1 Comparison of baseline data between the two groups

There were no statistically significant differences between the two groups in gender, age, BMI and segmental distribution (*P* > 0.05, **Table 1**).

**Table 1:** Comparison of baseline data of CSM patients in the Open-ACDF and Endo-ACDF groups.

Characteristic	Endo-ACDF group	Open-ACDF group	*P* value
Age (year)	52.24 ± 9.37	51.75 ± 10.16	0.372
Sex (M/F, n)	34/31	28/30	0.790
BMI (kg/m^2^)	25.23 ± 5.17	25.02 ± 4.63	0.817
Segment (n)			0.165
C_3/4_	6	5	
C_4/5_	31	18	
C_5/6_	20	29	
C_6/7_	8	6	

CSM, Cervical spondylotic myelopathy; ACDF, anterior cervical discectomy and fusion; Endo-ACDF, Spinal endoscopy assisted anterior cervical discectomy and fusion; BMI, Body mass index.

### 3.2 Comparison of surgical conditions between the two groups

Operative time was significantly shorter in the Open-ACDF group than in the Endo-ACDF group (*P* < 0.05). Additionally, intraoperative blood loss and postoperative drainage were significantly lower in the Endo-ACDF group compared with the Open-ACDF group (*P* < 0.05). No significant difference was observed in hospitalization time between the two groups (*P* > 0.05, **Table 2**). Serum CRP and CPK levels peaked on postoperative day 1 and gradually decreased to near-normal values by day 3, showing an initial increase followed by a decline. No significant difference in this trend was observed between the two groups (*P* > 0.05, **Supplementary Table S1**).

**Table 2:** Comparison of surgical conditions of CSM patients in the ACDF and Endo-ACDF groups.

Characteristic	Endo-ACDF group	Open-ACDF group	*P* value
Intraoperative estimated blood loss (mL)	27.31 ± 5.27	34.57 ± 6.15	0.037
Postoperative drainage (mL)	20.47 ± 4.31	29.83 ± 5.71	0.021
Operation time (min)	81.32 ± 18.43	73.47 ± 21.15	0.043
Hospital stay (day)	6.13 ± 1.36	6.27 ± 1.18	0.775

CSM, Cervical spondylotic myelopathy; Open-ACDF, Open anterior cervical discectomy and fusion; Endo-ACDF, Percutaneous full-endoscopic technique assisted anterior cervical discectomy and fusion.

### 3.3 Comparison of the JOA, NDI and VAS scores

At 1 week postoperatively, JOA, NDI, and VAS scores differed significantly between the two groups (*P* < 0.05); however, no significant differences were observed at 6 months, 12 months, or at the final follow-up (*P* > 0.05, **Table 3**).

**Table 3:** Comparative analysis of JOA, NDI, and VAS scores before and after the operation in both groups.

Time	Endo-ACDF group	Open-ACDF group	*P* value
JOA score			
Preoperative	7.03 ± 1.74	6.87 ± 1.25	0.824
Postoperative 1 week	13.13 ± 2.29*	10.07 ± 2.75*	0.013
Postoperative 6 months	13.35 ± 2.77*	13.54 ± 3.02*	0.902
Postoperative 12 months	14.11 ± 3.11*	14.04 ± 2.93*	0.927
Last follow-up	15.53 ± 3.27*	15.26 ± 3.75*	0.821
NDI (%)			
Preoperative	46.34 ± 14.27	45.75 ± 15.03	0.795
Postoperative 1 week	30.27 ± 10.36*	35.35 ± 12.04*	0.018
Postoperative 6 months	19.45 ± 4.35*	19.87 ± 5.12*	0.873
Postoperative 12 months	14.16 ± 3.34*	14.23 ± 3.19*	0.913
Last follow-up	11.87 ± 3.02*	12.03 ± 3.11*	0.883
VAS score			
Preoperative	7.23 ± 2.01	7.15 ± 1.93	0.871
Postoperative 1 week	3.52 ± 1.13*	4.13 ± 1.32*	0.027
Postoperative 6 months	2.18 ± 0.83*	2.21 ± 0.85*	0.931
Postoperative 12 months	1.68 ± 0.67*	1.71 ± 0.72*	0.813
Last follow-up	1.18 ± 0.71*	1.20 ± 0.68*	0.874

Open-ACDF, Open anterior cervical discectomy and fusion; Endo-ACDF, spinal endoscopy assisted anterior cervical discectomy and fusion; JOA, japanese orthopedic association; VAS, visual analog scale; NDI, neck disability index. *represents a significant difference to the preoperative value.

### 3.4 Comparison of the radiological outcomes

Both groups demonstrated significant improvements in CLA, HAVB, and ROM at 1 year postoperatively and at the final follow-up compared with preoperative values (*P* < 0.05). No significant differences in preoperative CLA, HAVB, or ROM were observed between the two groups (*P* > 0.05). At 1 year postoperatively, CLA and HAVB were significantly higher in the Endo-ACDF group than in the Open-ACDF group (*P* < 0.05). At the final follow-up, HAVB remained significantly higher in the Endo-ACDF group (P < 0.05), whereas no significant difference in CLA was observed between the two groups (*P* > 0.05). No statistically significant difference in postoperative ROM was observed between the two groups (*P*>0.05, **Table 4**).

**Table 4:** Comparative analysis of radiological outcomes before and after the operation in both groups.

Time	Endo-ACDF group (n=65)	ACDF group (n=58)	*P* value
CLA (°)			
Preoperative	8.38 ± 3.12	8.45 ± 2.95	0.921
Postoperative 1 year	14.81 ± 4.33*	13.17 ± 4.07*	0.031
Last follow-up	16.34 ± 4.16*	16.27 ± 4.29*	0.837
HAVB (mm)			
Preoperative	40.02 ± 8.93	41.13 ± 8.26	0.684
Postoperative 1 year	44.73 ± 8.25*	42.12 ± 9.01*	0.038
Last follow-up	44.15 ± 9.17*	42.31 ± 8.34*	0.025
ROM(°)			
Preoperative	40.36 ± 5.21	40.58 ± 6.13	0.893
Postoperative 1 year	42.74 ± 7.38*	42.59 ± 7.21*	0.902
Last follow-up	42.09 ± 6.73*	42.47 ± 6.48*	0.837
Bone graft fusion (%)\			
Postoperative 6 months	92.54	90.65	0.384
Postoperative 1 year	98.71	95.37	0.491

ACDF, anterior cervical discectomy and fusion; Endo-ACDF, spinal endoscopy assisted anterior cervical discectomy and fusion; CLA, cervical lordosis angle; HAVB, height of the adjacent vertebral body; ROM, range of motion; *represents a significant difference to the preoperative value.

At 6 months postoperatively, bone graft fusion was achieved in 92.54% of patients in the Endo-ACDF group and 90.65% in the Open-ACDF group. By the 1-year follow-up, fusion rates increased to 98.71% and 95.37% in the Endo-ACDF and Open-ACDF groups, respectively. Statistical analysis revealed no significant difference between the two groups at either the 6-month or 1-year follow-up (*P* > 0.05, **Table 4**).

### 3.5 Complications

In the Endo-ACDF group, one patient (1.54%) developed postoperative cervical axial pain, which was relieved with conservative treatment using nonsteroidal anti-inflammatory drugs (NSAIDs). Two patients (3.08%) developed localized subcutaneous edema, which was alleviated with diuretic therapy. One patient (1.54%) experienced mild dysphagia, which gradually resolved with symptomatic treatment during the follow-up period.

In the Open-ACDF group, two patients (3.45%) developed transient dysphagia, three patients (5.17%) developed localized hematomas, and two patients (3.45%) experienced postoperative neck pain. All these complications were effectively managed with symptomatic treatment during follow-up. One patient (1.72%) developed adjacent segment disease (ASD), which was successfully treated with percutaneous endoscopic anterior cervical discectomy. No significant difference was observed in the incidence of postoperative complications between the two groups (*P* > 0.05, **Table 5**).

**Table 5:** Comparative analysis of complications in both groups.

Group	Dysphagia	Cervical axial pain	Localized edema	ASD and revision	Total
Endo-ACDF group	1	1	2	0	4
ACDF group	2	2	3	1	8
*P* value					0.154

ACDF, anterior cervical discectomy and fusion; Endo-ACDF, spinal endoscopy assisted anterior cervical discectomy and fusion; ASD, adjacent segment disease.

## 4 Discussion

ACDF is considered the “gold standard” for the treatment of radiculopathy and CSM, producing satisfactory postoperative outcomes (17–19). Emami et al. (20) treated 205 patients with CSM using ACDF, reporting a postoperative complication rate of 2.9% and a revision rate of 7.8% over a minimum follow-up of 7 years. Despite its advantages, ACDF has limitations, including a limited surgical workspace, unclear anatomical landmarks, and incomplete posterior decompression of the vertebral body, which may increase the risk of complications. In recent years, spine surgeons have explored new techniques to assist ACDF in the treatment of CSM and enhance its effectiveness. Currently, microscope-assisted ACDF and endoscopy-assisted ACDF are commonly employed in clinical practice for the treatment of CSM (21, 22). Spinal endoscopic techniques, initially applied to lumbar degenerative diseases, have now been extended to the treatment of cervical and thoracic degenerative diseases (13, 23, 24). Compared with the naked eye or microscope view, water-mediated spinal endoscopy provides a clearer operative field (25, 26). It enables precise hemostasis and allows clear identification and meticulous removal of osteophytes at the posterior margin of the vertebral body (27).

Currently, there are limited reports on spinal endoscopy-assisted ACDF for the treatment of cervical degenerative diseases. Yao et al. (9) performed ACDF with single-channel endoscopic assistance in patients with cervical disc herniation. Among 67 patients with a minimum follow-up of 5 years, 86.6% achieved satisfactory surgical outcomes. However, due to the absence of postoperative MRI analysis and lack of a control group, the authors concluded that their findings require further validation. Ahn et al. (28) reported favorable clinical outcomes for cervical disc herniation treated with spinal endoscopic techniques over a 5-year follow-up period. However, spatial constraints of the operating channel restricted visual exposure, limited the use of surgical instruments, and hindered fusion device implantation. Moreover, endoscopic manipulation under continuous water medium may result in complications, including neck swelling, mediastinal effusion, and spinal cord hypertension (29). In our previous study, unilateral biportal endoscopy (UBE) was used to assist the ACDF procedure, combining the advantages of both air and water media. This approach offers a clear surgical field, facilitates instrument maneuvering, and allows thorough decompression, leading to favorable surgical outcomes (30). However, due to inherent limitations of UBE, the viewing and working channels are separated, making manipulation of larger laminar and nucleus pulposus forceps relatively difficult. Therefore, we further refined the technique and opted for single-channel Endo-ACDF.

The results of this study demonstrated that postoperative JOA, NDI, VAS, CLA, HAVB, and ROM were significantly improved compared with preoperative values in both groups. These findings suggest that both Open-ACDF and Endo-ACDF can achieve thorough decompression, provide stable fixation, and accomplish final osseous fusion, resulting in comparable clinical outcomes. At 1 year postoperatively, CLA was significantly greater in the Endo-ACDF group than in the Open-ACDF group. This improvement is primarily attributed to the coaxial spinal endoscope’s capability to access the posterior margin of the vertebral body, allowing more precise management of the posterior longitudinal ligament and osteophytes. Such meticulous management reduces the likelihood of postoperative ossification, which could otherwise cause local stiffness and impede CLA improvement (31). Furthermore, a reduction in HAVB increases bony pressure on the cervical nerve roots. This nerve root irritation induces pain, prompting a compensatory reduction in CLA to relieve nerve root compression (32, 33).

The Endo-ACDF group exhibited significantly lower intraoperative estimated blood loss and postoperative drainage compared with the Open-ACDF group. During Endo-ACDF, spinal endoscopy provides clear visualization of the venous plexus, facilitating rapid and accurate identification of bleeding sites and allowing effective hemostasis with bipolar electrocoagulation, thereby preventing postoperative intravertebral hematoma formation. Spinal endoscopy provides clear illumination and high-resolution visualization, significantly reducing the risk of injury to the spinal cord, dura mater, and nerve roots, and enhancing surgical precision compared with traditional surgery. Clinical results suggest that adjunctive spinal endoscopic techniques enhance the safety and efficacy of ACDF without increasing surgical risks, while substantially reducing the risk of nerve root and dural sac injury. To objectively assess the invasiveness of Endo-ACDF and Open-ACDF, muscle damage was evaluated by measuring preoperative and postoperative changes in serum CPK and CRP levels. In both groups, CPK and CRP levels exhibited an initial elevation followed by a gradual decline. Comparative analysis indicated that although CPK and CRP levels were generally higher in the Open-ACDF group than in the Endo-ACDF group, these differences were not statistically significant. These findings suggest that the invasiveness of Endo-ACDF is comparable to that of Open-ACDF.

Open-ACDF and Endo-ACDF do not exhibit absolute superiority; their primary differences relate to surgical trauma, operative conditions, recovery efficiency, and suitable clinical indications. Open-ACDF provides a clear surgical field, ample working space, and a well-established technique, making it particularly appropriate for complex pathologies, including multilevel disc herniation and severe osteophyte formation. However, it is associated with relatively greater surgical trauma, prolonged postoperative recovery, prominent neck scarring, and a higher incidence of axial pain. Endo-ACDF employs spinal endoscopy, resulting in minimal intraoperative bleeding, reduced postoperative pain, accelerated recovery, and concealed scarring. It is particularly suitable for single-level lesions and patients prioritizing cosmetic outcomes. However, it requires higher technical proficiency and entails increased costs due to specialized instruments. Clinical decision-making necessitates comprehensive assessment of the affected segment, lesion severity, and the surgeon’s technical proficiency. Key outcome measures, including long-term fusion rates and the incidence of adjacent segment degeneration, still require validation through extended follow-up studies.

The indications and contraindications for Endo-ACDF are largely consistent with those of Open-ACDF, with certain limitations: (1) The procedure involves a relatively steep learning curve, necessitating substantial experience in open surgery and proficiency in spinal endoscopic techniques. (2) Owing to the setup of the endoscopic fixation system and the learning curve, operative time may be slightly prolonged.

Successful Endo-ACDF relies on precise operative technique and meticulous handling (34). Key preoperative precautions include accurate small-incision localization using imaging and body surface marking to prevent soft tissue traction injuries. The endoscope should be introduced after exposing the intervertebral space (during Casper retractor insertion) and removed following fusion cage placement. Prepare 0° lenses for deep structure visualization and 30° lenses for a wide field, allowing on-demand interchange. Hemostasis should be managed by controlling blood pressure (systolic 90–100 mmHg; mean arterial pressure 60–70 mmHg), addressing venous plexus bleeding with radiofrequency or gelatin sponges, using bone wax-coated drills for cancellous bone bleeding, and considering preoperative tranexamic acid administration. Perfusion pressure should be maintained below 30 mmHg, avoiding pressure elevation for hemostasis. Select 2–4 mm grinding heads, avoid long-axis drills, and ensure that drill working components remain under endoscopic visualization and within the intervertebral space to prevent soft tissue entanglement.

The main innovations of this procedure include: (1) Spinal endoscopes provide operational flexibility and eliminate the need for repeated lens adjustments, thereby expanding the surgical field, improving efficiency, and minimizing blind spots. (2) The procedure is performed in an open rather than a confined environment, maintaining a clear operative field via water flow without increasing water pressure. This strategy reduces the risk of high pressure on the spinal cord and prevents postoperative fluid accumulation and edema in the loose soft tissues of the cervical region. (3) The procedure preserves coaxial alignment between the viewing and working channels, effectively preventing obstruction of the visual field by surgical instruments during operation.

## 5 Limitations of this study

This study has several limitations. Being a retrospective study, it cannot be compared with double-blind studies regarding the choice of surgical modality, and it is also prone to bias due to subjective factors. As a new technology, continuous improvement of endoscopic fixation devices and surgical instrumentation is necessary, and further large-scale, multicenter studies are needed to further evaluate its efficacy and safety. While Endo-ACDF demonstrated advantages in intraoperative hemostasis and visualization, the specific contributions of water-mediated endoscopy to long-term alignment and neurological recovery warrant further investigation through prospective, controlled studies.

## 6 Conclusions

Endo-ACDF combines the endoscopic system with ACDF technology for treating CSM, demonstrating clinical efficacy comparable to Open-ACDF. Compared to Open-ACDF, Endo-ACDF offers a clearer surgical field, improved intraoperative hemostasis, and reduced intraoperative blood loss and postoperative drainage.

## Data Availability

The datasets presented in this study can be found in online repositories. The names of the repository/repositories and accession number(s) can be found in the article/**Supplementary Material**.
