# Supplementary material for: Early efficacy and safety of spinal endoscopy assisted anterior cervical discectomy and fusion in the treatment of cervical spondylotic myelopathy
Source: Front Oncol. 2026 Feb 18;16:1678009. doi: 10.3389/fonc.2026.1678009 (PMC12956701; doi:10.3389/fonc.2026.1678009)
Supplement: Supplementary file 1 [file DataSheet1.docx]

**Supplementary Images**


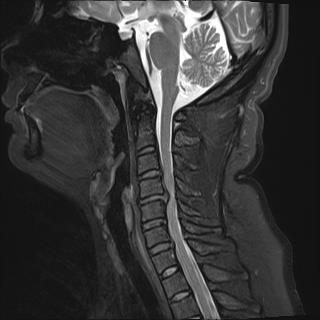


Preoperative MRI of C4/5 in a patient with cervical spondylotic myelopathy demonstrates spinal cord compression caused by the intervertebral disc. (sagittal)


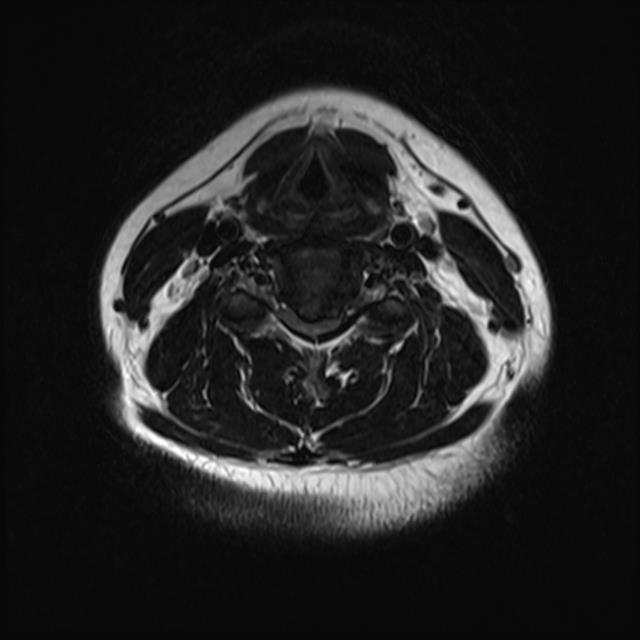


Preoperative MRI of C4/5 in a patient with cervical spondylotic myelopathy demonstrates spinal cord compression caused by the intervertebral disc. (axial position)


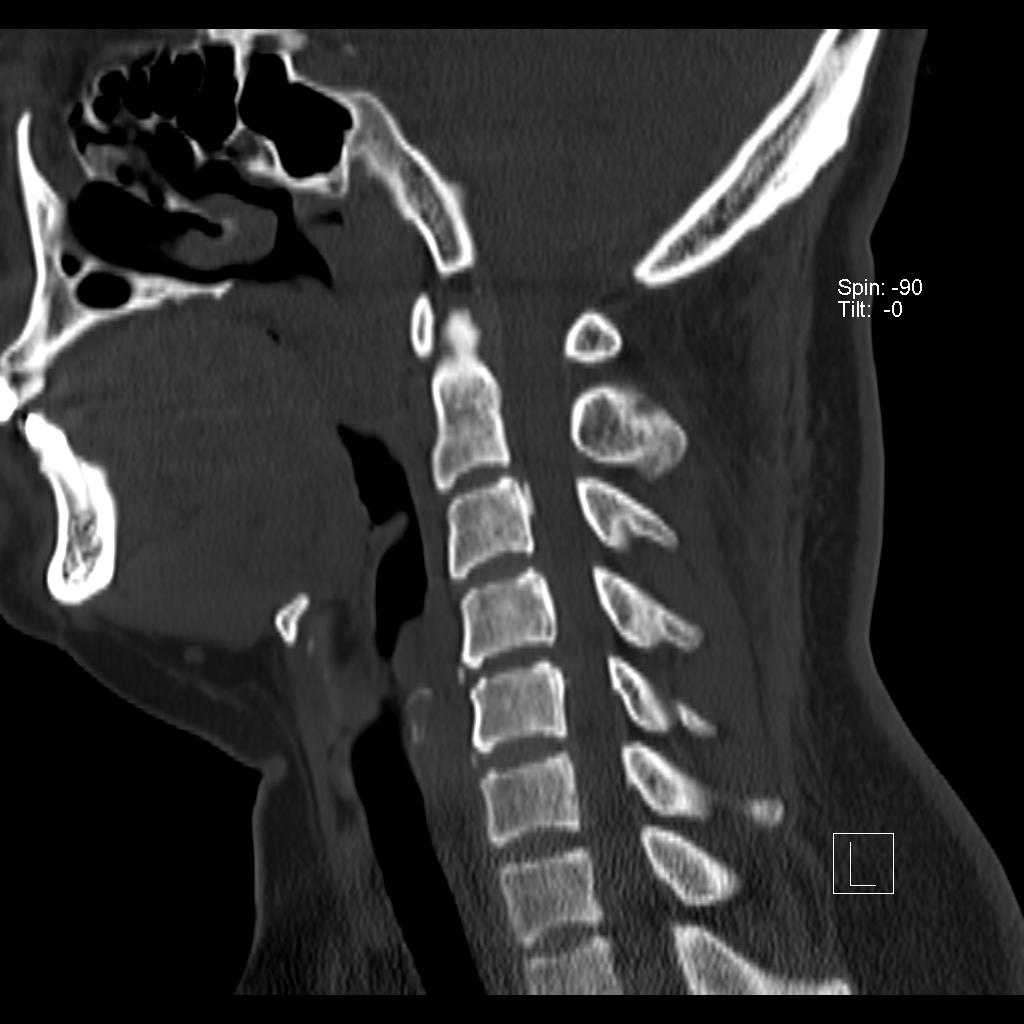


Preoperative CT of C4/5 in a patient with cervical spondylotic myelopathy demonstrates spinal cord compression caused by the intervertebral disc. (sagittal)


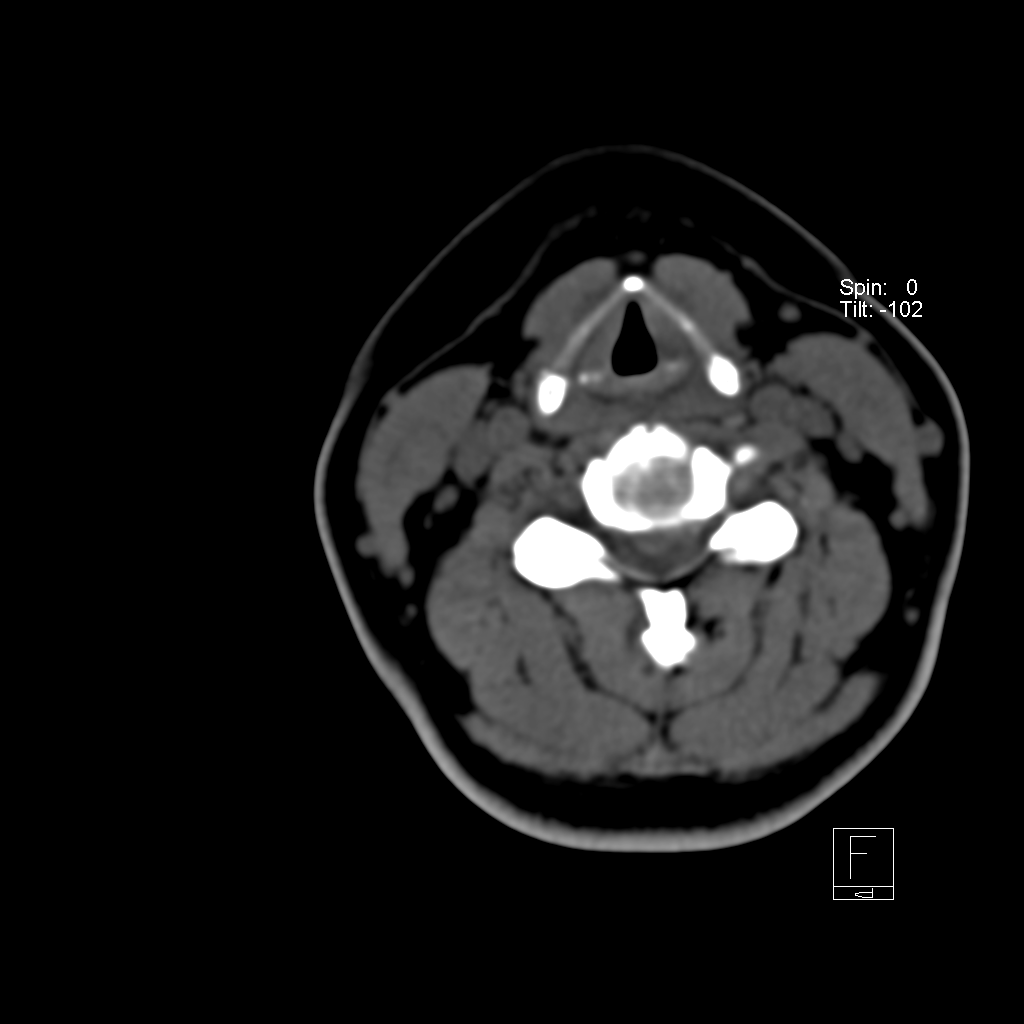


Preoperative CT of C4/5 in a patient with cervical spondylotic myelopathy demonstrates spinal cord compression caused by the intervertebral disc. (axial position)


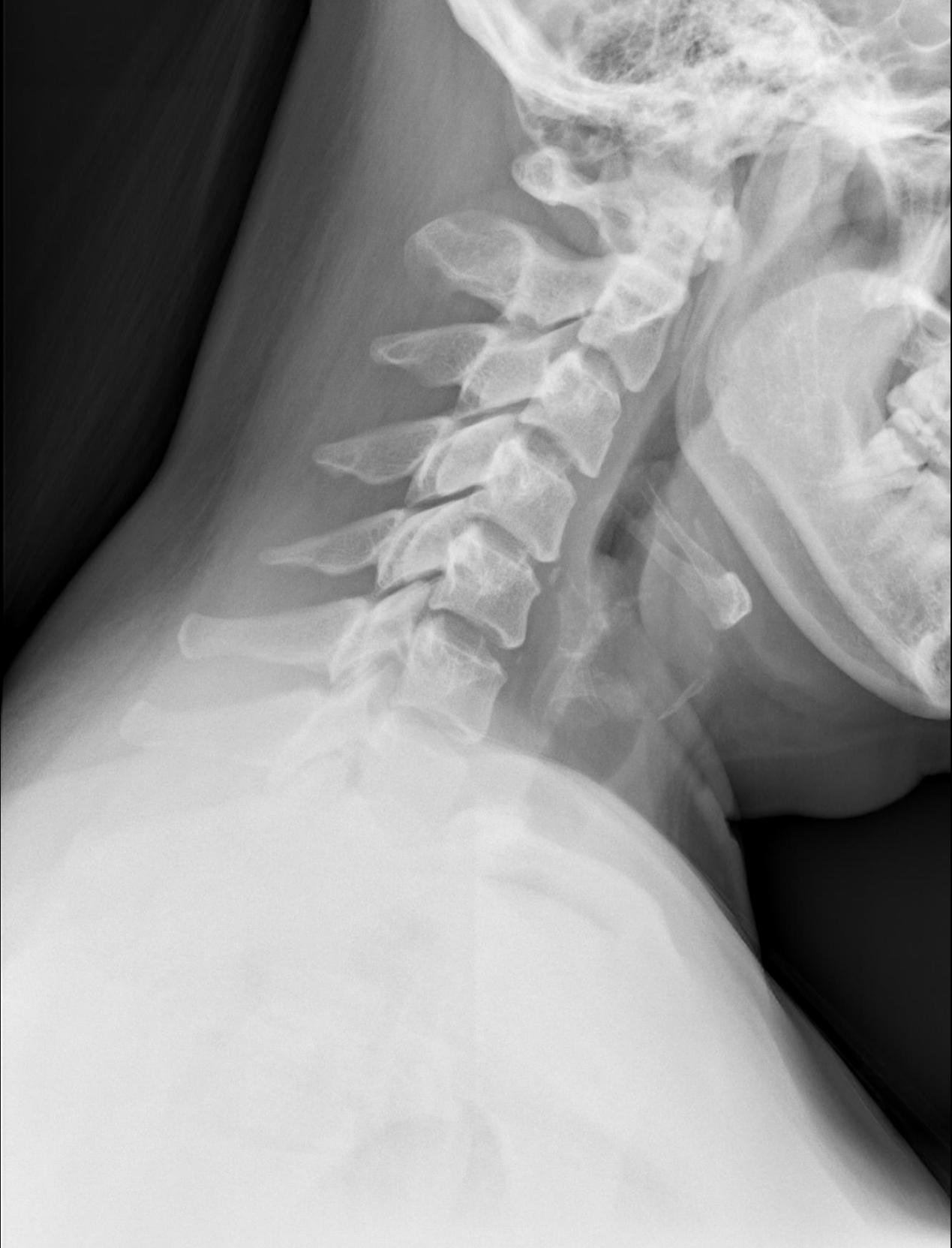


Preoperative DR


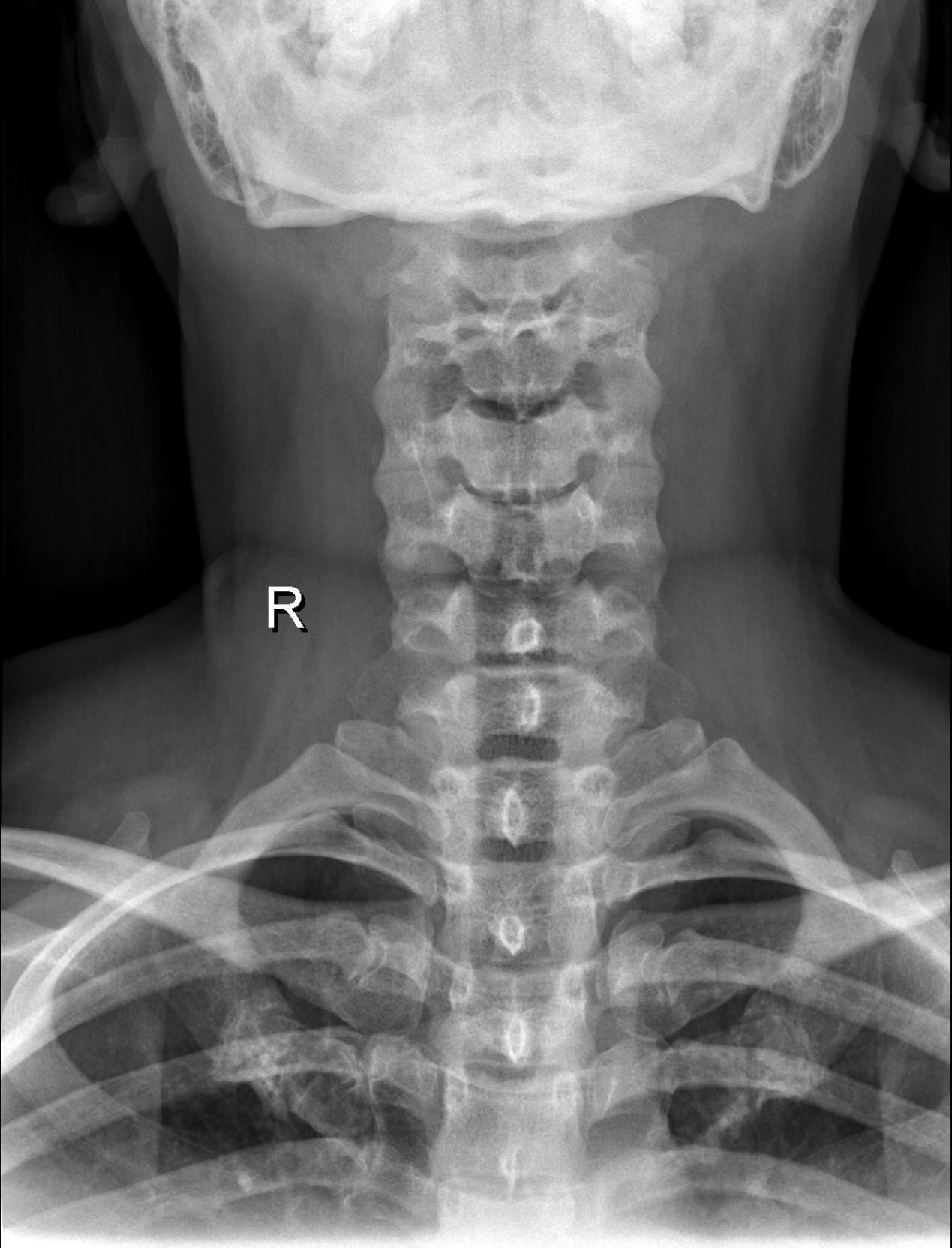


Preoperative DR


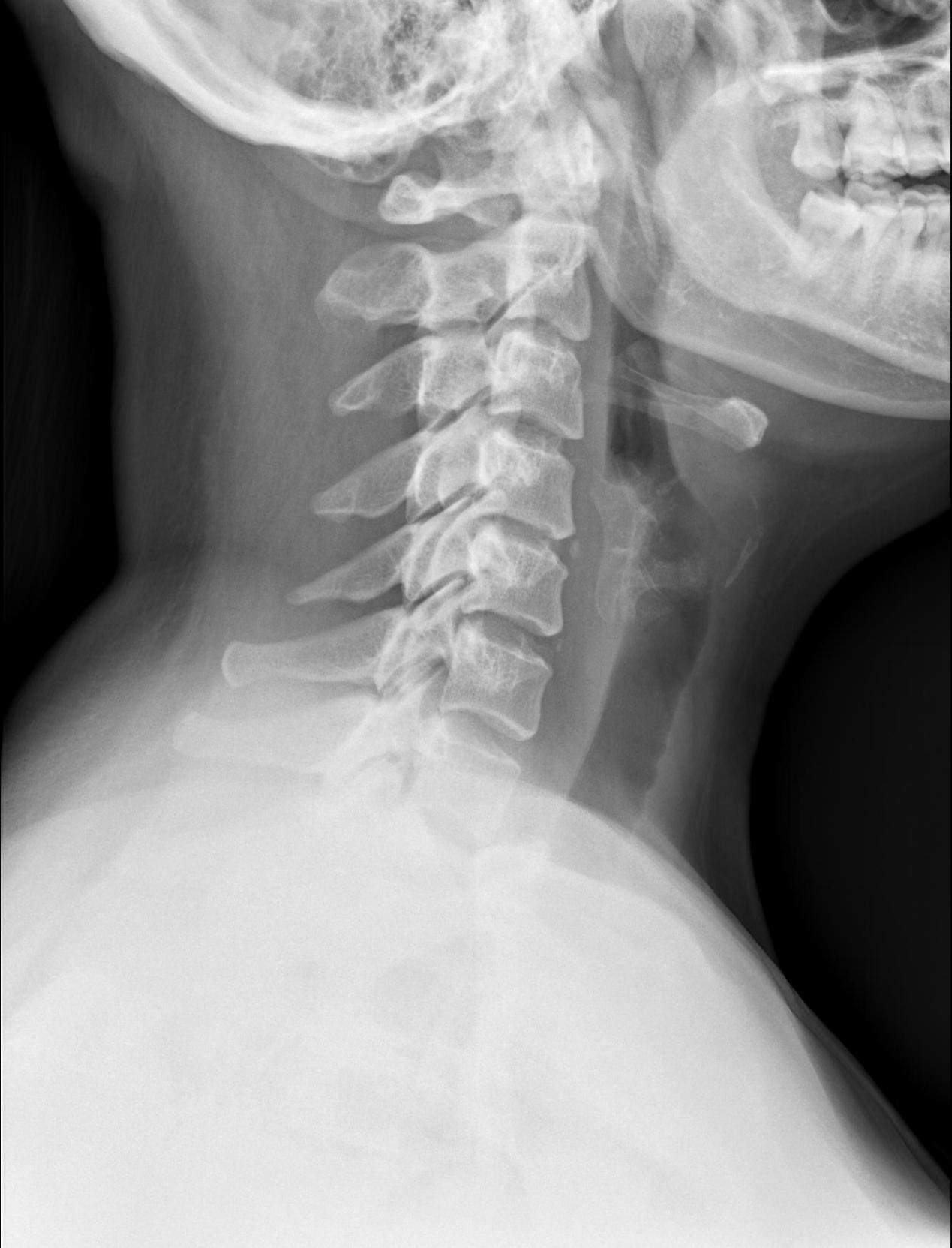


Preoperative DR


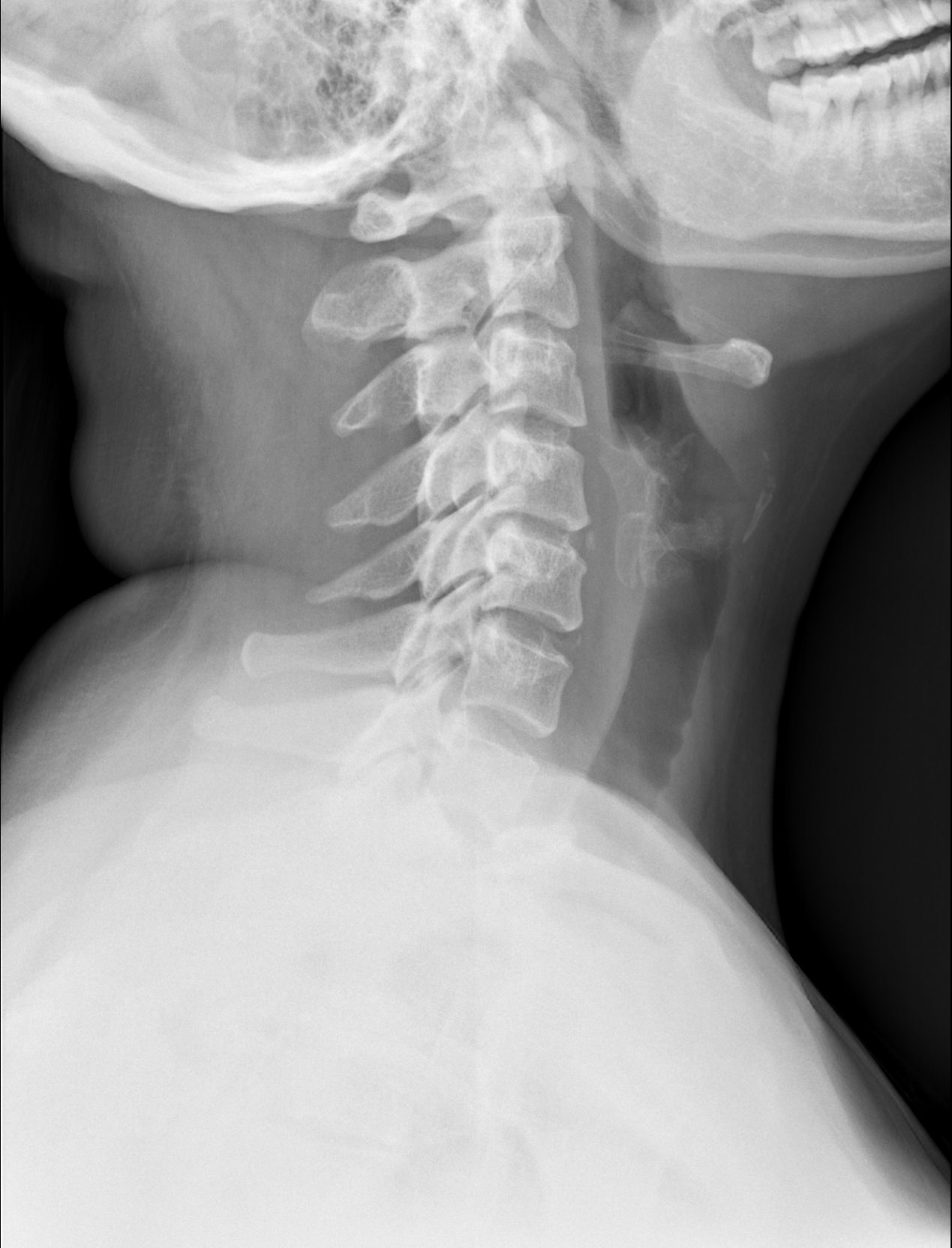


Preoperative DR


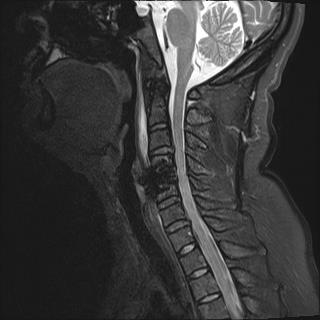


Postoperative sagittal MRI of C4/5 in a patient with cervical spondylotic myelopathy demonstrates relief of spinal cord compression.
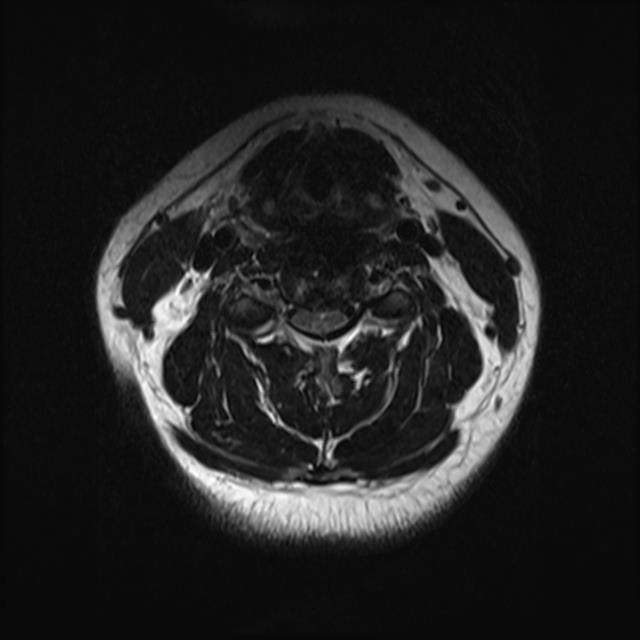


Postoperative axial MRI of C4/5 in a patient with cervical spondylotic myelopathy demonstrates relief of spinal cord compression.


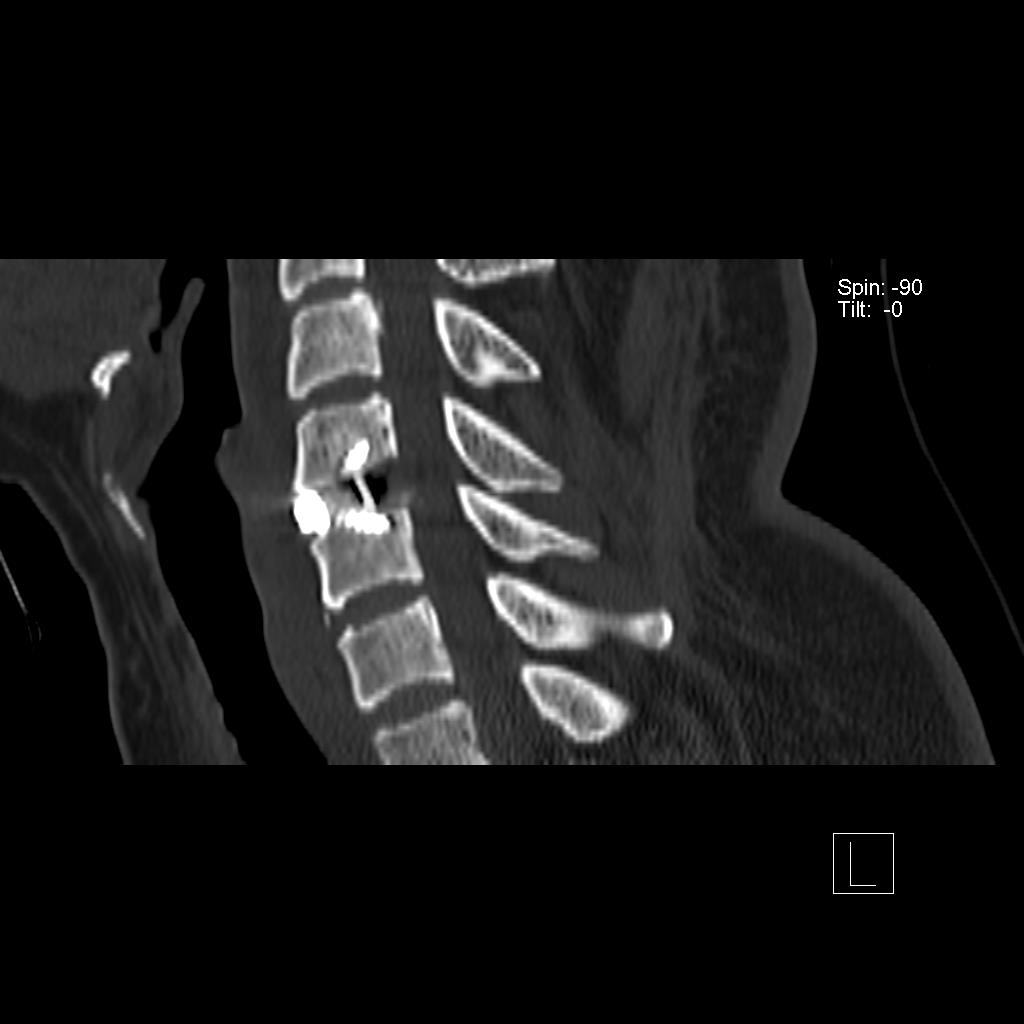


Postoperative sagittal CT of C4/5 in a patient with cervical spondylotic myelopathy demonstrates relief of spinal cord compression.


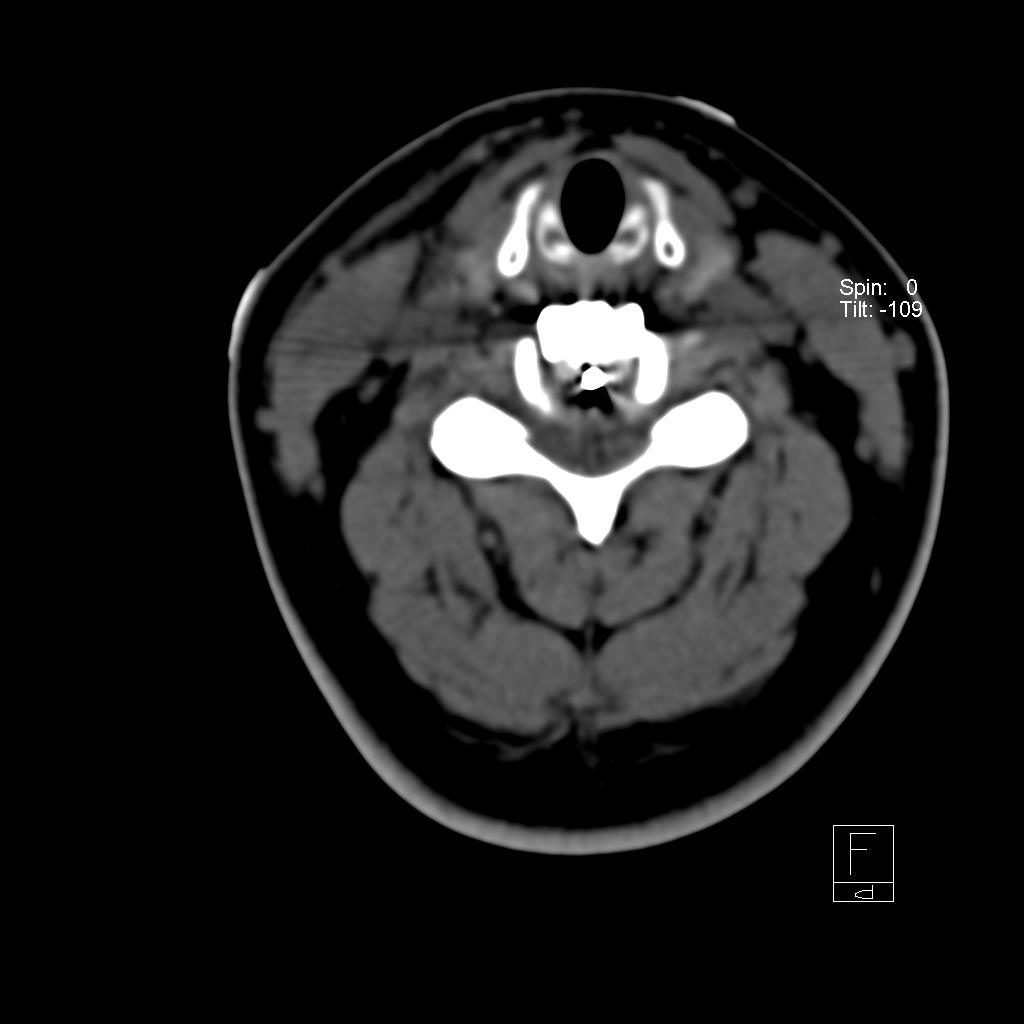


Postoperative axial CT of C4/5 in a patient with cervical spondylotic myelopathy demonstrates relief of spinal cord compression.


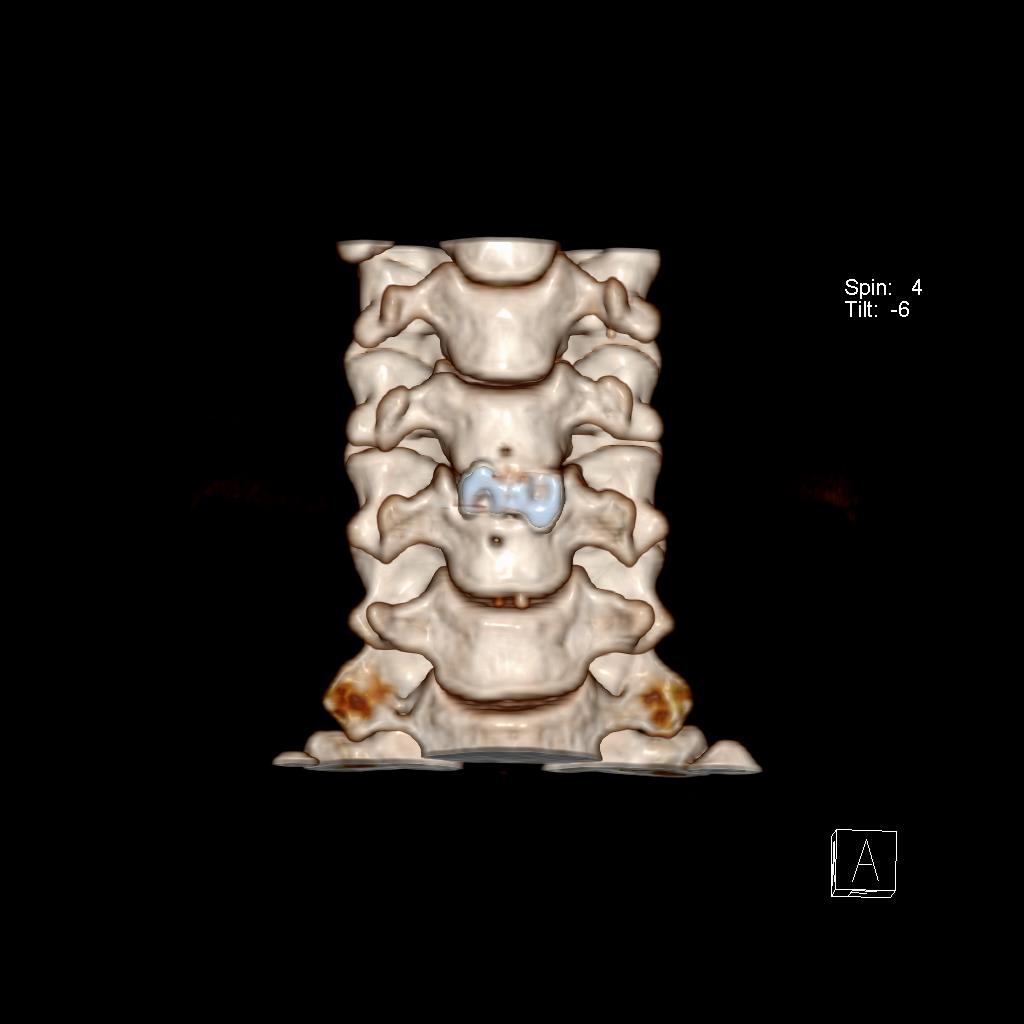


Postoperative three-dimensional CT reconstruction demonstrates that the fusion device is properly positioned.


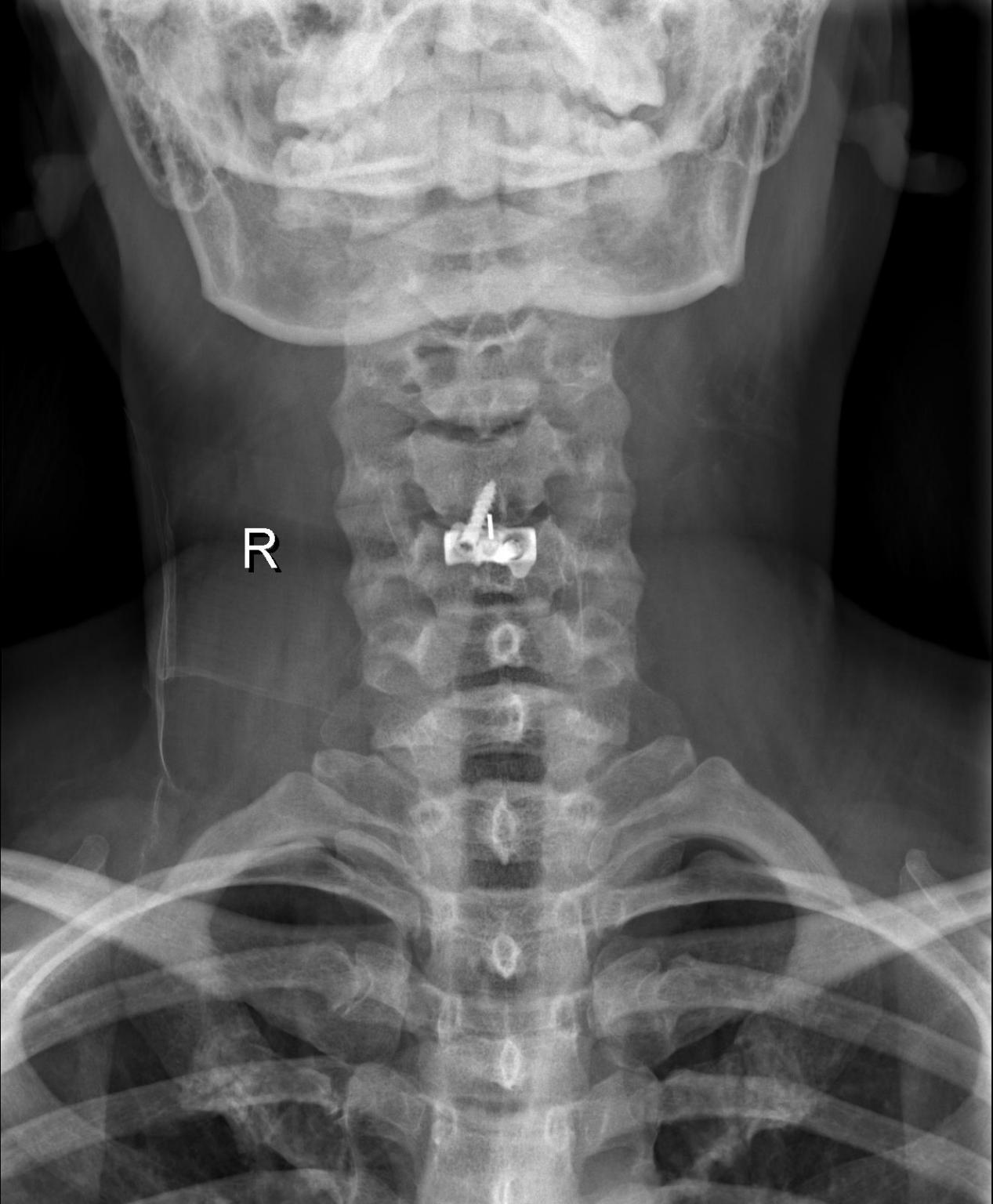


Postoperative DR reconstruction demonstrates that the fusion device is properly positioned.


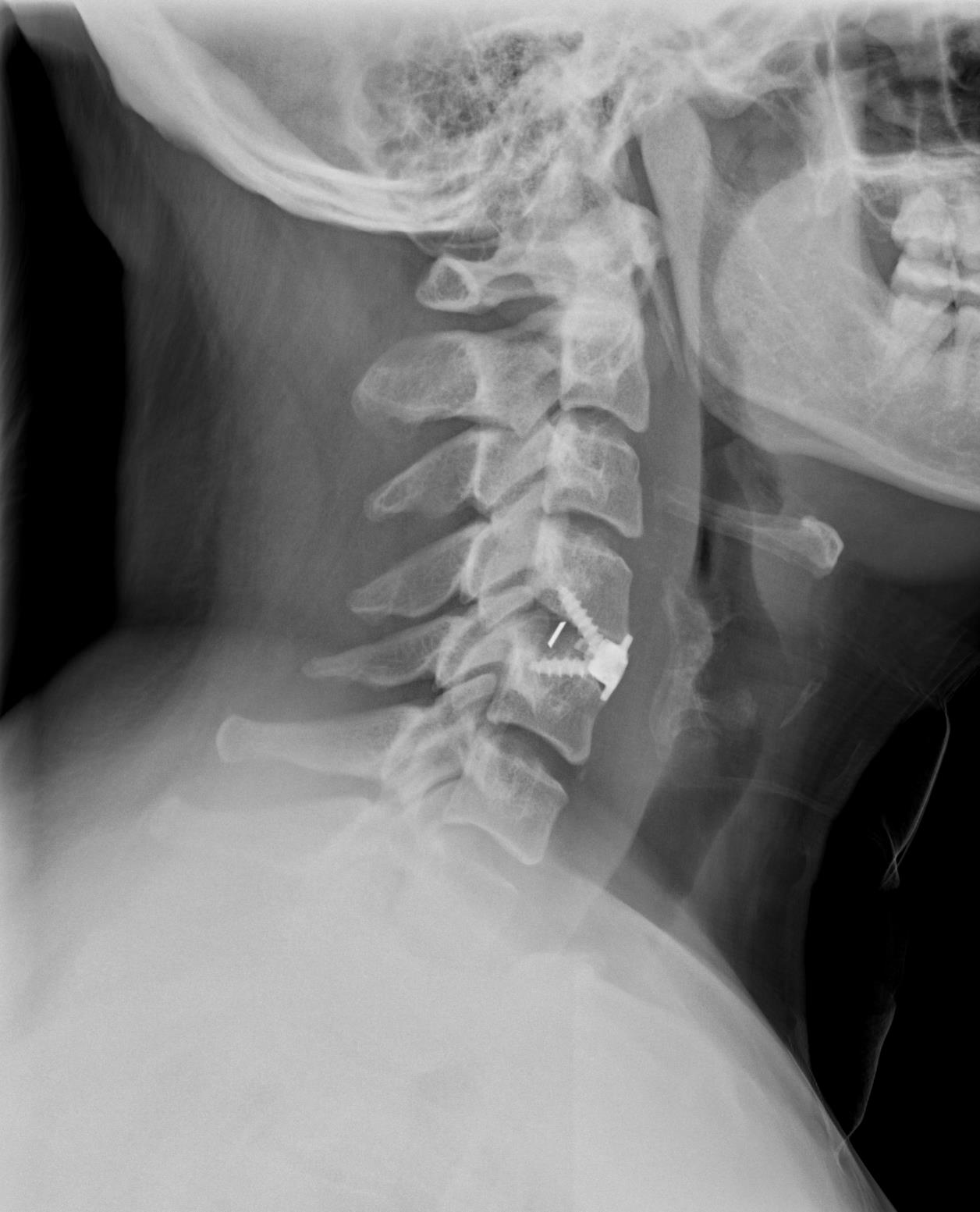


Postoperative DR reconstruction demonstrates that the fusion device is properly positioned.
