# Supplementary material for: Early efficacy and safety of spinal endoscopy assisted anterior cervical discectomy and fusion in the treatment of cervical spondylotic myelopathy
Source: Front Oncol. 2026 Feb 18;16:1678009. doi: 10.3389/fonc.2026.1678009 (PMC12956701; doi:10.3389/fonc.2026.1678009)
Supplement: Supplementary file 2 [file Table1.docx]

**Supplementary Table 1** Comparison of CRP and CPK levels between the ACDF and Endo-ACDF groups.

| Characteristic | Endo-ACDF group | Open-ACDF group | *P* value |
| --- | --- | --- | --- |
| CRP (mg/dL) |  |  |  |
| Preoperative | 0.17±0.04 | 0.23±0.05 | 0.732 |
| Postoperative 1 day | 0.43±0.21 | 0.39±0.25 | 0.605 |
| Postoperative 3 days | 0.15±0.08 | 0.17±0.10 | 0.920 |
| CPK (IU) |  |  |  |
| Preoperative | 98.24±28.45 | 102.87±31.05 | 0.645 |
| Postoperative 1 day | 139.78±51.89 | 131.47±57.72 | 0.325 |
| Postoperative 3 days | 103.21±49.05 | 101.76±52.17 | 0.741 |

CRP, C-reactive protein, CPK, Creatine phosphokinase.
